# Capsazepine decreases corneal pain syndrome in severe dry eye disease

**DOI:** 10.1186/s12974-021-02162-7

**Published:** 2021-05-11

**Authors:** Darine Fakih, Adrian Guerrero-Moreno, Christophe Baudouin, Annabelle Réaux-Le Goazigo, Stéphane Mélik Parsadaniantz

**Affiliations:** 1 Sorbonne Université, INSERM, CNRS, Institut de la Vision, 17 rue Moreau, F-75012 Paris, France; 2 R&D Department, Laboratoires Théa, 12 rue Louis Biérot, F-63000 Clermont-Ferrand, France; 3 CHNO des Quinze-Vingts, INSERM-DGOS CIC 1423, 17 rue Moreau, F-75012 Paris, France; 4 Department of Ophthalmology, Ambroise Paré Hospital, AP-HP, University of Versailles Saint-Quentin-en-Yvelines, 9 avenue Charles de Gaulle, F-92100 Boulogne-Billancourt, France

**Keywords:** Trigeminal pain, Nociceptors, Electrophysiology, Dry eye, Behavior, TRPV1 antagonist

## Abstract

**Background:**

Dry eye disease (DED) is a multifactorial disease of the ocular surface accompanied by neurosensory abnormalities. Here, we evaluated the effectiveness of transient receptor potential vanilloid-1 (TRPV1) blockade to alleviate ocular pain, neuroinflammation, and anxiety-like behavior associated with severe DED.

**Methods:**

Chronic DED was induced by unilateral excision of the Harderian and extraorbital lacrimal glands of adult male mice. Investigations were conducted at 21 days after surgery. The mRNA levels of TRPV1, transient receptor potential ankyrin-1 (TRPA1), and acid-sensing ion channels 1 and 3 (ASIC1 and ASIC3) in the trigeminal ganglion (TG) were evaluated by RNAscope in situ hybridization. Multi-unit extracellular recording of ciliary nerve fiber activity was used to monitor spontaneous and stimulated (cold, heat, and acid) corneal nerve responsiveness in ex vivo eye preparations. DED mice received topical instillations of the TRPV1 antagonist (capsazepine) twice a day for 2 weeks from d7 to d21 after surgery. The expression of genes involved in neuropathic and inflammatory pain was evaluated in the TG using a global genomic approach. Chemical and mechanical corneal nociception and spontaneous ocular pain were monitored. Finally, anxiety-like behaviors were assessed by elevated plus maze and black and white box tests.

**Results:**

First, in situ hybridization showed DED to trigger upregulation of TRPV1, TRPA1, ASIC1, and ASIC3 mRNA in the ophthalmic branch of the TG. DED also induced overexpression of genes involved in neuropathic and inflammatory pain in the TG. Repeated instillations of capsazepine reduced corneal polymodal responsiveness to heat, cold, and acidic stimulation in ex vivo eye preparations. Consistent with these findings, chronic capsazepine instillation inhibited the upregulation of genes involved in neuropathic and inflammatory pain in the TG of DED animals and reduced the sensation of ocular pain, as well as anxiety-like behaviors associated with severe DED.

**Conclusion:**

These data provide novel insights on the effectiveness of TRPV1 antagonist instillation in alleviating abnormal corneal neurosensory symptoms induced by severe DED, opening an avenue for the repositioning of this molecule as a potential analgesic treatment for patients suffering from chronic DED.

## Background

Dry eye disease (DED) is a multifactorial disease of the ocular surface and tears accompanied by neurosensory abnormalities [1]. Numerous symptoms, such as redness, burning, itching, and pain, have been observed in DED patients [2, 3]. In addition, clinical data have shown that DED is often associated with anxiety and depression syndromes [4, 5]. To date, the management of chronic corneal pain still represents an important therapeutic challenge.

The cornea is the most densely innervated tissue in the body [6, 7]. Corneal nociceptive innervation is provided by ciliary nerves originating from the ophthalmic branch of the trigeminal ganglion (TG) [8, 9]. Different corneal nociceptors coexist at a single sensory nerve ending [10]. Approximately 40% are polymodal nociceptors, sensitive to heat, acidity, and chemical agents; 50% are cold thermoreceptors; and 10% are mechano-nociceptors [11]. Their sensory modalities are linked to the specific expression of ion channels: transient receptor potential vanilloid-1 (TRPV1), TRP ankyrin1 (TRPA1), and acid-sensing ion channels (ASICs 1 and 3) for polymodal nociceptors; TRP melastatin 8 (TRPM-8) for cold thermoreceptors; and piezo-type mechanosensitive ion channel component 2 (Piezo-2) for mechano-nociceptors [10].

Electrophysiological studies of trigeminal neurons in DED models have shown increased nociceptor responsiveness to cold and heat stimulation [12] and corneal nerve endings to acidic and thermal stimulation [13]. Moreover, a TRPV1 antagonist (capsazepine) prevents dry eye sensitization of cold-sensing cells to capsaicin [14]. TRPV1, which is gated by capsaicin, protons, and noxious heat [15], can be activated by hypertonic challenge, causing increased pro-inflammatory cytokine production, underlining its involvement in DED [16, 17]. Increased TRPV1 protein levels in the TG and their role in enhanced nocifensive behavior has been reported for DED rats [18]. In addition, TRPV1 inhibition reduced polymodal responsiveness to acidic stimulation in an allergic eye model [19]. TRPV1 pharmacological blockade decreases substance P release in cold allodynia [20], and DED sensitizes corneal cold nociceptive neurons via TRPV1 [14]. Finally, ocular instillation of tivanisiran, a small interfering oligonucleotide of RNA designed to silence human TRPV1, has been shown to improve ocular hyperemia and tear quality in humans [21].

We aimed to investigate the pharmacological effectiveness of topical TRPV1 antagonist treatment (capsazepine) on abnormal corneal neurosensitivity associated with persistent DED. We used our recently published preclinical mouse model of chronic DED induced by excision of the extraorbital lacrimal gland (ELG) and Harderian gland (HG) [22]. First, we studied polymodal and mechano-nociceptor mRNA expression in the ophthalmic branch of the TG under conditions of DED. Second, we evaluated the spontaneous and evoked electrical activity of the corneal nerves in response to heat, cold, and acid stimulation. Next, we assessed the effectiveness of topical treatment with capsazepine (twice a day for 21 days) on corneal nerve activity in response to heat, cold, and acid stimulation. Finally, we evaluated the beneficial effects of such treatment on nociceptive and anxiety behaviors associated with DED and on the modulation of the expression of genes involved in neuropathic and inflammatory pain in TG.

## Methods

### Experimental animals

Seven- to 8-week-old adult male C57BL/6 mice (average weight 23.48 ± 0.04 g) (Janvier Labs, Le Genest Saint Isle, France) were randomly assigned to cages (5 mice/cage) and maintained under controlled conditions (22 ± 1 °C, 60 ± 10% relative humidity, 12/12 h light/dark cycle, food and water ad libitum). All animal procedures were performed in strict accordance with institutional guidelines for the care and use of experimental animals approved by the European Communities Council Directive 2010/63/UE (APAFIS #1501 2015081815454885 v2). A well-being unit in accordance with ethics guidelines followed all experiments.

### Surgical procedures

Unilateral (right side) ELG and HG excision was performed under ketamine (80 mg/kg intraperitoneal (i.p.)) and xylazine (8 mg/kg i.p.) anesthesia in mice, as recently described [22]. Before surgery, a drop of lacrimal gel (Lubrithal™, Dechra) was applied to both eyes. Under an operative microscope (Leica-Alcon II, Germany), an 8-mm skin incision was made on the temporal side to expose and remove the ELG. After dissociating the conjunctival tissue above the orbital cavity near the internal canthus, the HG was carefully removed. Complete removal was verified by inspecting the surgical area for any remaining glandular tissue. The skin incision was then sutured using 6.0 braided silk sutures (6-0 Vicryl, Ethicon, Scotland). A drop of iodine solution was applied to the incision to avoid bacterial infection. For sham animals, an incision was made in the same zone without touching the glands. The mice were placed in warm (30 °C) cages to recover from the surgery.

### Drugs

Capsazepine (Sigma-Aldrich) was initially dissolved in 99.8% methanol and diluted 1:1325 in sterile endotoxin-free isotonic phosphate-buffered saline (PBS) to obtain a final concentration of 10 μM. Capsaicin (Sigma-Aldrich) was dissolved in 100% ethanol and then diluted 330X in sterile endotoxin-free isotonic PBS to obtain a 100 μM solution. The vehicle-treated group corresponded to mice treated with sterile endotoxin-free isotonic PBS containing the same proportion of ethanol or methanol as the final capsazepine or capsaicin dilutions, respectively.

### Therapeutic strategy

The topical ocular treatments (vehicle, capsazepine) were randomly assigned to each cage before surgery (sham or DED). Topical ocular instillation, which was only performed in the right eye, with solvent or 10 μM capsazepine started at d7 post-surgery and continued until d21. DED animals were treated twice a day (10 am and 5 pm) in their home cages. Moreover, the cage order was randomized daily for topical ocular administration. All tests were conducted on d21. Behavioral tests were conducted first (eye closing ratio, von Frey, and anxiety). For behavioral tests, mice were placed in the testing room (at least 30–60 min before the start of the experiments), and the order of testing was randomized. The investigator was blinded to the treatment. The animals were then divided into two groups, one for electrophysiological experiments and the other for the measurement of mRNA levels in the TG. Electrophysiological traces were analyzed in a blinded manner, as the experimenter was blinded to the treatment, as well as the group of mice analyzed: operated (sham, DED) or naïve (WT).

For microscopic examination, c-Fos immunostaining was performed on the amygdala of mice from the sham and DED experimental groups in the absence of any eye stimulus; a single investigator analyzed all data in a blinded manner. Analyses were performed only on the right eye and ipsilateral TG.

### Behavioral tests

#### Measurement of corneal sensitivity to mechanical and chemical stimulation

Mechanical corneal sensitivity was monitored using von Frey filaments as previously described [22]. Various forces of calibrated von Frey filaments (0.008 to 0.04 g) were applied to the center of the cornea of manually immobilized mice. The mechanical threshold corresponded to the eye-blink response. For corneal chemical sensitivity, 10 μl of 100 μM capsaicin was applied to the right eye. Animals were immediately placed in individual cages, and the palpebral closure time of the right eye was measured for 5 min.

#### Anxiety tests

Before the tests, mice were acclimated to the experimental room for a minimum of 60 min. We used a video-tracking system and analyzed behavioral parameters with the Smart 3 software (Harvard Apparatus). The floor and walls of the testing box were cleaned between animal tests with 70% ethanol to avoid any perturbation.

##### Elevated plus maze

The animal was placed in the center area of the elevated plus maze (Bioseb in vivo research instruments, France) with its head directed toward a closed arm. Time spent in the open arms (s) was recorded for 5 min [23]. The behavioral parameters were analyzed using the Smart 3 software (Harvard Apparatus).

##### Black and white test (light-dark box test)

The animal was placed in the middle of a brightly illuminated chamber of the dark-light box device (Bioseb in vivo research instruments). The time spent in the white zone (%) was recorded for 5 min [24] using the Smart 3 software (Harvard Apparatus).

##### Measurement of the eye-closing ratio

Spontaneous eye closure is an accurate index for monitoring spontaneous eye pain [22] and is among the quantitative measures of the grimace scale, which is used to monitor spontaneous pain behavior [25, 26]. The eye closing ratio was measured for the right eye based on photographs of mice that were awake and unconstrained and corresponds to the height/width ratio of palpebral closure. The width is the distance between the internal and external canthus and the height, the distance between the edge of the upper and lower eyelids, going through the center of the cornea. Images were captured by a digital camera using the EyeSuite™ software (Koeniz, Switzerland).

#### Multi-unit extracellular recording of spontaneous and stimulated activities of ciliary nerve fibers in ex vivo eye preparations

Spontaneous ciliary nerve fiber activity was determined at d21 as previously reported [22, 27]. Briefly, mice were euthanized, and the right eye (corresponding to the side of the surgery) rapidly and carefully dissected and placed in a two-compartment chamber. The baseline recordings were performed by superfusing the cornea with superfusion saline solution at 33 ± 1°C and pH 7.4. The evoked activity of the ciliary nerve was measured in response to acid or thermal stimulation. Acid stimulation was carried out by changing the pH of the superfusion saline solution from 7.4 to 6 and then 5. Thermal stimulation consisted of modifying the temperature of the superfusion saline solution from 33 ± 1 down to 20 ± 1 °C (cold stimulations) or up to 40 ± 1 °C (heat stimulations) at pH 7.4.

Multi-unit extracellular electrical activity was recorded from the ciliary nerve (composed of a large number of nerve fibers) using a suction electrode (Ag/AgCl). The signal was filtered (300–5000 Hz), amplified (×10,000) (A-M Systems, Sequim, USA), and digitalized by Spike 2 data analysis (CED Micro1401, Cambridge Electronic Design) at a sampling frequency of 10,000 Hz. The cornea was superfused with superfusion saline solution for 30 min to stabilize the preparation before performing the electrophysiological recordings. Spontaneous extracellular ciliary nerve fiber activity was defined as impulses per second (imp/s).

A thermistor sensor (included in the CL-100 Bipolar Temperature Controller, Warner Instruments) monitored the temperature at the exit of the corneal superfusion. Chemical stimulation was achieved by exposing the cornea to a CO_2_ (100%) pulse for 30 s.

### RT-qPCR analysis

#### Tissue preparation for RT-qPCR analysis

Twenty-one days after surgery, the animals were deeply anesthetized with a 300-μL mixture of ketamine (80 mg/kg) and xylazine (8 mg/kg) and transcardially perfused with 10 mL 0.9% NaCl solution. The ipsilateral TG (corresponding to the eye with LG excisions) was rapidly and carefully dissected and placed at –80°C until use.

#### RT-qPCR analysis protocol

RNA extraction from the ipsilateral TG was performed using a NucleoSpin RNA Purification II kit (NucleoSpin RNA S, Germany). RNA quality and concentration were then measured by the NanoDrop method (Thermo Scientific, England). Then, reverse transcription was performed using the iScript cDNA Synthesis Kit (Bio-Rad) according to the manufacturer’s instructions. PCR was performed with 300 ng cDNA for each sample. RT-qPCR was performed using SsoAdvanced Universal SYBR® Green supermix (Bio-Rad) and a pain, neuropathic, and inflammatory (SAB Target List) M96-well plate (Bio-Rad; ref 10034393). The GAPDH gene was used as the endogenous reference for each reaction; mRNA levels were calculated after normalizing the results for each sample with those for GAPDH mRNA. The 2-ΔΔCt method was used to analyze the relative differences in specific mRNA levels between groups.

### Tissue preparation for fluorescent in situ hybridization and immunostaining

Twenty-one days after surgery, anesthetized mice were transcardially perfused with 10 mL 0.9% NaCl solution followed by 40 mL 4% (w/v) paraformaldehyde in 1X PBS. Next, TGs were immersed in 10%, 20%, and 30% sucrose in 1X PBS and then conserved in isopentane with liquid nitrogen and stored at –80 °C. Brain (12 μm) and TG (12 μm) thin sections were cut using a cryostat (Leica CM 3050 S) and mounted on Superfrost slides.

### Fluorescent in situ hybridization protocol

Fluorescent in situ hybridization studies were performed according to the protocol for fixed frozen tissue using the RNAscope Fluorescent Multiplex Reagent kit v2 assay (Advanced Cell Diagnostics, Newark, CA, USA). Tissues were washed with 1X PBS and treated with hydrogen peroxide (RNAscope, ref 322335) for 10 min at room temperature and washed in autoclaved distilled water. Using a steamer, tissues were treated with distilled H_2_O for 10 s at 99°C and then moved to RNAscope 1X target Retrieval Reagent (RNAscope, ref# 322000) for 5 min at 99°C. Tissues were washed with autoclaved distilled water and transferred to 100% alcohol for 3 min. Then, tissues were treated with RNAscope Protease III (RNAscope, ref 322337) for 30 min at 40°C and washed with autoclaved distilled water. Species-specific target probes TRPV1 C1 (313331 C1), TRPA1 C2 (400211 C2), Piezo-2 C3 (400191 C3), ASIC1 C2 (462381 C2), and ASIC3 C3 (480541 C3) were used. Sections were treated with the probe and negative (ref 320871) and positive (ref 320881) controls and hybridized for 2 h at 40°C in a humidified oven (RNAscope HybEZ oven with HybEZ humidity control tray, Advanced Cell Diagnostics). A series of incubations was then performed to amplify the signal of the hybridized probe and label target probes for the assigned fluorescence detection channel (target probe was labeled for the assigned fluorescence detection channels Opal 520 FP1487001, Opal 570 FP1488001, and Opal 650 FP1496A, PerkinElmer). Nuclei were stained using a DAPI nuclear stain (RNAscope ref 323108) for 30 s at room temperature. The sections were finally mounted with ProLong Gold Antifade Mountant (ref P36934) onto glass sides and coverslipped.

### cFOS immunostaining in the amygdala

Brain sections were rinsed in 0.1M PBS with 3% H_2_O_2_. Then, brain sections were incubated for 1 h in a blocking solution of 0.1M PBS containing 3% normal goat serum and 0.1% triton X-100, followed by incubation with the primary antibody polyclonal guinea pig anti-c-Fos (Synaptic Systems, ref 226 004, 1:1000) at 4 °C for 72 h. All steps following incubation with the primary antibody were performed at room temperature (RT).

Next, brain sections were rinsed in 0.1M PBS-T and incubated with biotinylated goat anti-guinea pig secondary antibody (Vector, ref BA-7000-1.5, 1:500) for 1 h at RT. Samples were washed in PBS and incubated with VECTASTAIN® Elite® ABC HRP (PK-6100, Vector, A reagent 1:250 and B reagent 1:250) in PBS for 1 h at RT. Tissues were rinsed with PBS and then 0.05 M Tris buffer. Revelation was performed in 0.003% H_2_O_2_ with 0.05% 3,3′-diaminobenzidine (DAB) in 0.05 M Tris buffer for 11 min. Frozen sections were dehydrated in an ethanol series at 50%, 70%, 90%, and 100% and SaveSolv (VWR Q-Path) for 10 min each. Finally, the sections were mounted onto glass slides with Eukitt mounting medium (Sigma Aldrich) and coverslipped.

### Microscopic and NanoZoomer analysis

Brain and ipsilateral TG sections were examined using a NanoZoomer 2.0-HT digital slide scanner (C9600, Hamamatsu Photonics). The area within the field of interest covered by the mRNA profiles relative to the total area of the measured field was analyzed to detect mRNA levels in the ophthalmic branch of the TG. The same gray threshold level was applied to all sections of the same series. For c-FOS staining, the number of positive cells in the amygdala was quantified. TIFF images were analyzed using the NIH ImageJ software.

### Statistical analysis

The data obtained for different groups were compared using the appropriate paired parametric or nonparametric statistical test, as indicated. For statistical analysis, the Kolmogorov–Smirnov test was performed followed by parametric *t*-tests or nonparametric Mann-Whitney or nonparametric Kruskal-Wallis tests using GraphPad Prism version 7.00 (GraphPad Software, La Jolla, CA, USA). All *P* values were considered statistically significant for values < 0.05. All results are presented as the mean ± standard error of the mean (SEM). The unit of analysis was single mouse for all experiments.

## Results

### Chronic DED increases nociceptor mRNA levels in the ophthalmic branch of the TG

**Fig. 1:**
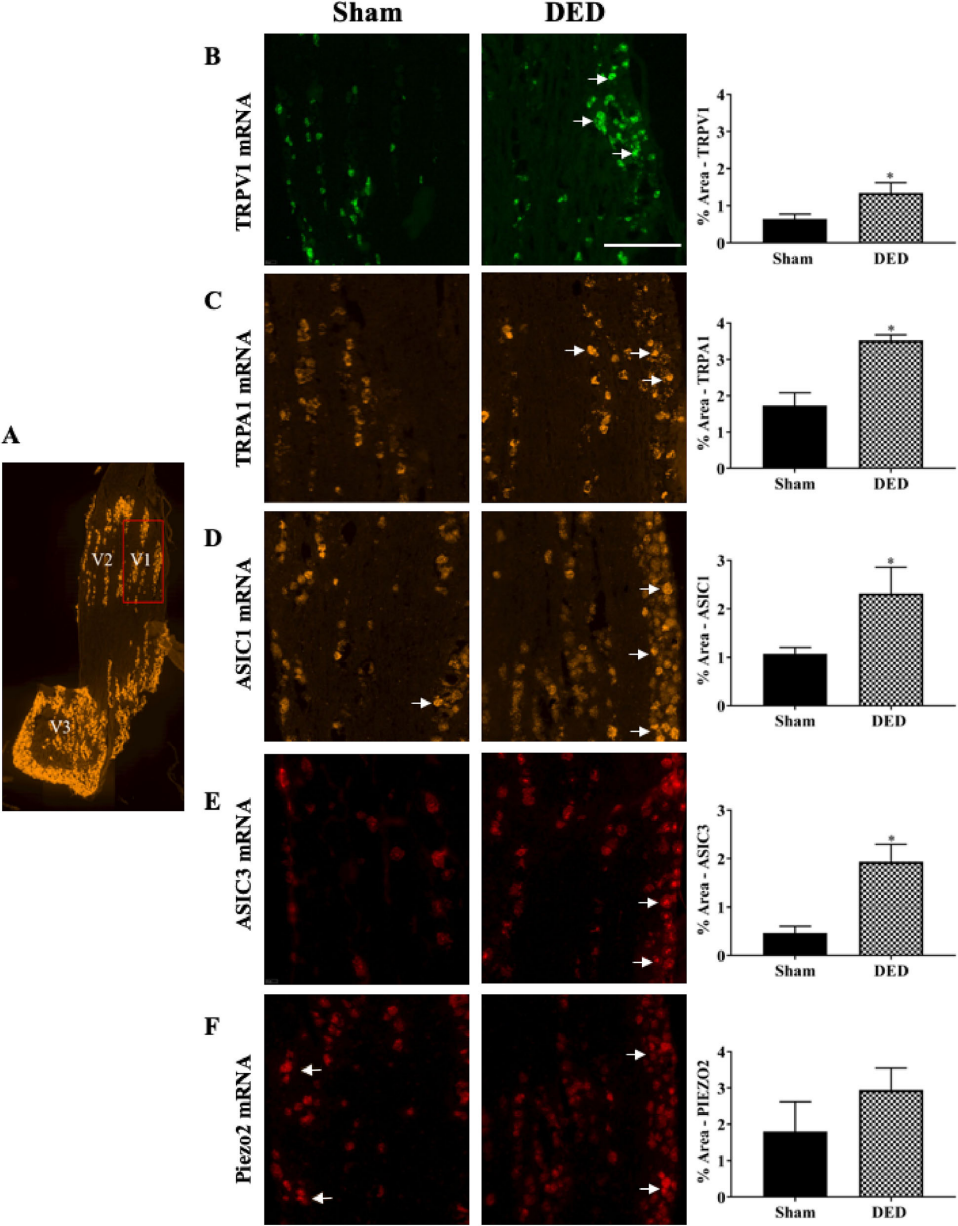
Evaluation of nociceptor expression in the ophthalmic branch of the trigeminal ganglion (TG) of sham and dry eye disease (DED) animals. **a** Localization of the ophthalmic branch (V1, red rectangle), maxillary branch (V2), and mandibular branch (V3) in a mouse TG using the positive probe of RNAscope (Opal 520). Staining (white arrows) and quantification of mRNA levels by in situ hybridization of (**b**) transient receptor potential vanilloid-1 (TRPV1; Opal 520), (**c**) transient receptor potential ankyrin 1 (TRPA1; Opal 570), (**d**) acid-sensing ion channel 1 (ASIC1; Opal 570), (**e**) ASIC3 (Opal 650), and (**f**) Piezo-2 (Opal 650) in the ophthalmic branch of the TG of sham and DED animals. All experiments were conducted on d21. Total number of mice = 44. Sham and DED animals *n* = 4–6. Scale bar = 50 μM. *P < 0.05 relative to sham group. Results are expressed as the mean ± SEM. For statistical analysis, the Kolmogorov–Smirnov test was performed followed by a parametric *t*-test (TRPV1) or nonparametric Mann-Whitney test (TRPA1, ASIC1, ASIC3, and Piezo-2) using GraphPad Prism version 7.00 (GraphPad Software, La Jolla, CA, USA)

We first evaluated the levels of TRPV1, TRPA1, ASIC1, ASIC3, and Piezo-2 mRNA in the ophthalmic branch of the TG of DED and sham animals at d21 by in situ RNA scope hybridization. TRPV1 and TRPA1 mRNA levels in the ophthalmic branch of DED animals were higher than those of sham animals (Fig. 1b, c; white arrows). The percentage of surface stained for TRPV1 and TRPA1 mRNA was 109.38% and 104.07% higher for DED than sham mice, respectively (TRPV1 0.64 ± 0.13 vs. 1.34 ± 0.27, TRPA1 1.72 ± 0.35 vs. 3.51 ± 0.15, *P* < 0.05; Fig. 1b, c). In addition, ASIC1 and ASIC3 mRNA staining in the ophthalmic branch of DED animals was 116.82% and 319.57% higher for DED than sham mice, respectively (ASIC1 1.07 ± 0.13 vs. 2.32 ± 0.53; ASIC3 0.46 ± 0.13 vs. 1.93 ± 0.36, *P* < 0.05; Fig. 1d, e; white arrows). However, Piezo-2 mRNA surface staining showed no significant differences between the two groups (1.79 ± 0.81 vs. 2.94 ± 0.60, *P* > 0.05, Fig. 1f, white arrows).

### Chronic DED induces chemical and mechanical corneal hypersensitivity and anxiety-like behavior

**Fig. 2:**
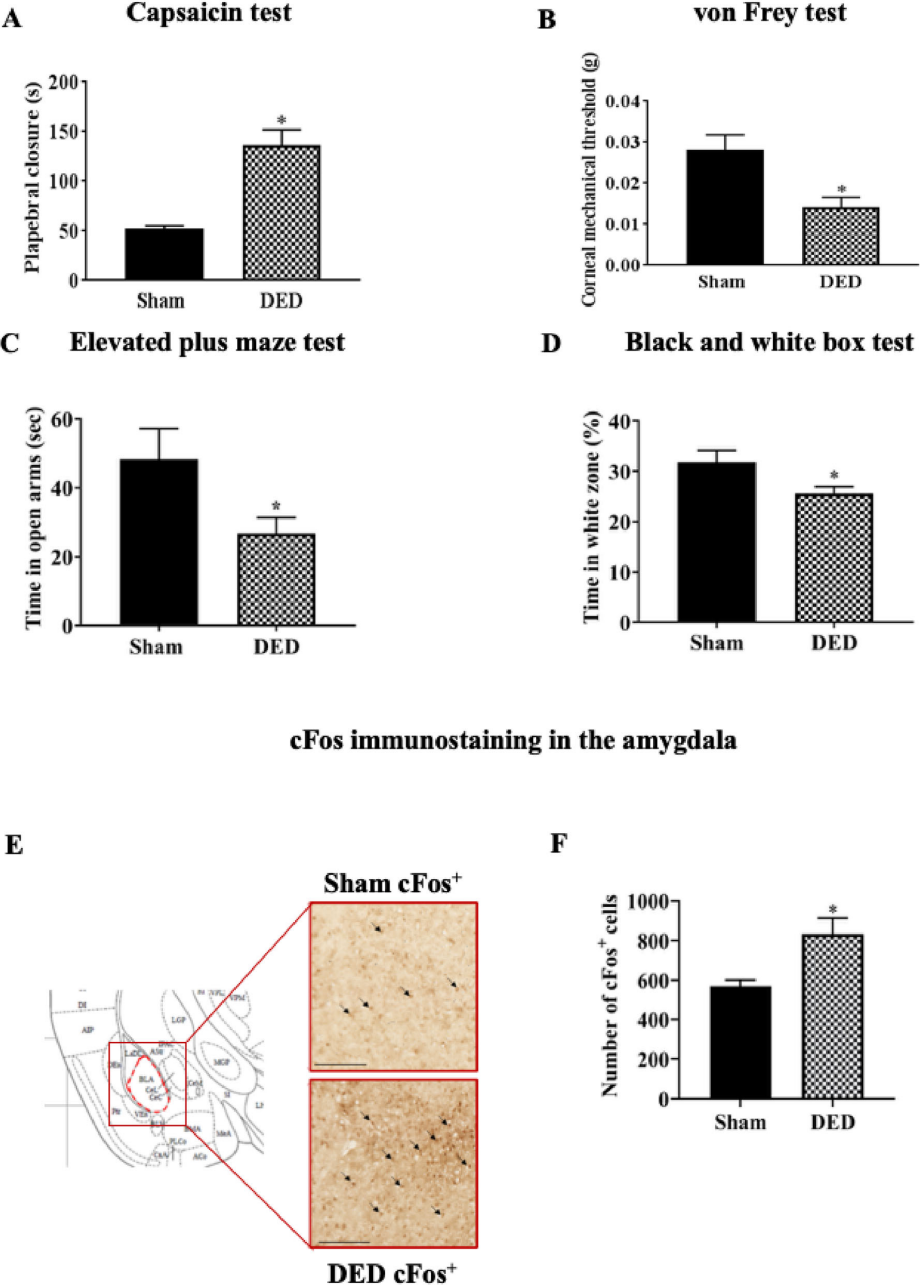
Evaluation of chemical and mechanical corneal sensitivity and anxiety-like behavior of sham and DED animals on d21. **a** Chemical corneal sensitivity was evaluated using a drop of capsaicin (100 μM) and recording the palpebral closure time for 5 min. **b** The corneal mechanical threshold was measured using von Frey filaments. **c** Elevated plus maze: the mouse was placed in the center area of the maze with its head directed toward a closed arm and time (s) in the open arms was recorded for 5 min. **d** Black and white test: The mouse was placed in the middle of a brightly illuminated chamber and time in the white zone (%) was recorded for 5 min. **e** cFos immunoreactivity in the amygdala of sham and DED mice; cFos^+^ cells (black arrows). Scale bar = 100 μm. Regions corresponding to the images are depicted in coronal diagrams taken from the Paxinos atlas [28]. **f** Quantification of the number of cFos^+^ cells in the amygdala of sham and DED animals. Total number of mice = 48. Number of mice included in the analysis = 48. Capsaicin and von Frey test: *n* = 6 sham and DED animals for each test. Elevated plus maze and black and white tests: *n* = 15 sham and DED animals; cFos immunoreactivity in amygdala: *n* = 3 sham and DED animals. *P < 0.05 relative to the sham group. Results are expressed as the mean ± SEM. For statistical analysis, the Kolmogorov–Smirnov test was performed followed by a parametric *t*-test (capsaicin test, von Frey test, elevated plus maze test, and black and white test) or nonparametric Mann-Whitney test (quantification of the number of cFos^+^ cells in the amygdala) using GraphPad Prism version 7.00 (GraphPad Software, La Jolla, CA, USA)

We evaluated corneal chemical sensitivity using the capsaicin test, which consists of the topical application of 100 μM capsaicin (TRPV1 agonist). The palpebral closure time increased by 159% in DED mice over that of sham mice (52.62 ± 2.78 s vs. 136.25 ± 15.46 s, *P* < 0.05, Fig. 2a). The corneal mechanical threshold measured with von Frey filaments was 50% lower for the DED than sham mice (0.028 ± 0.003 g vs. 0.014 ± 0.002 g, *P* < 0.05, Fig. 2b).

We examined the relationship between anxiety and DED-associated pain using two behavioral tests. First, the elevated plus maze test was used to evaluate the time spent in the open arms of the maze. DED mice spent approximately 44.5% less time in the open arms than sham mice (48.25 ± 8.88 s vs. 26.78 ± 4.64 s, *P* < 0.05, Fig. 2c). Then, the black and white test was used to measure the time spent in the white zone. DED mice spent approximately 18% less time in the white zone than sham mice (31.25 ± 2.33 s vs. 25.55 s ± 1.39, *P* < 0.05, Fig. 2d).

Preclinical and clinical studies suggest an important role for the amygdala in the development of chronic anxiety and pain [29, 30]. Thus, we explored changes in cFOS immunoreactivity (a marker of neuronal activation) in this brain structure. We observed greater cFOS immunostaining (black arrows) in the amygdala of DED than sham mice (Fig. 2e). The number of cFos-positive cells in the amygdala was 39% higher in the DED than sham animals (596.91 ± 17.90 cFOS-positive cells vs. 832.72 ± 46.10 cFOS-positive cells, *P* < 0.05, Fig. 2f).

### Chronic DED increases corneal nerve responsiveness

#### Cold and heat stimulation

**Fig. 3:**
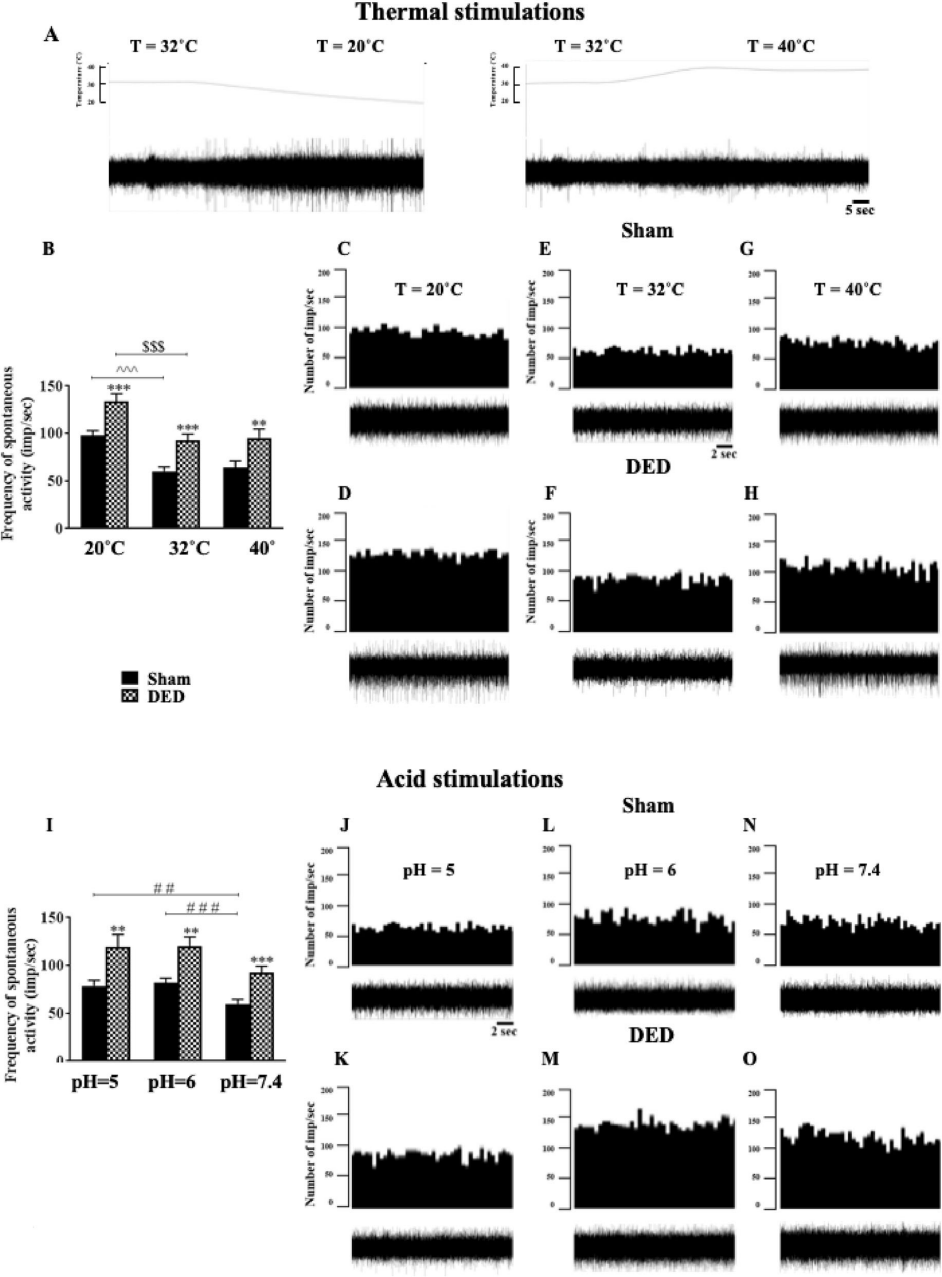
Ex vivo evaluation of spontaneous and evoked activities of the ciliary nerve from sham and DED mice. **a** Electrophysiological trace illustrating the extracellular activity of the ciliary nerve fiber with the various heat ramps; time scale = 5 s. Histograms showing the mean value of spontaneous (32°C) and evoked (20°C and 40°C) firing frequency of ciliary nerves: **b** at 20, 32, and 40°C. Representative histograms and electrophysiological traces to illustrate extracellular activity of the ciliary nerve: **c**, **d** at 20°C; **d**, **f** at 32°C; and **g**, **h** at 40°C in sham and DED mice; time scale = 2 s. Histograms showing the mean value for the spontaneous firing frequency of ciliary nerves: **i** at pH 5, 6, and 7.4 in both groups. Representative histograms and electrophysiological traces to illustrate extracellular activity of the ciliary nerve: **j**, **k** at pH 5; **l**, **m** at pH 6; and **N**, **O** at pH 7.4 in sham and DED mice; time scale = 2 s. All experiments were conducted on d21. Total number of mice = 27. Number of mice included in the analysis = 26. Thermal stimulation: *n* = 13 sham and DED animals; acid stimulation: *n* = 10 sham and DED animals. All experiments were conducted on d21. **P < 0.01, ***P < 0.001 relative to the sham group. ^^^ < 0.001 sham at 20°C and 32°C. $$$ < 0.001 DED at 20°C and 32°C. ## < 0.01 sham at pH 5 and 7.4. ### < 0.001 sham at pH 6 and 7.4. Results are expressed as the mean ± SEM. For statistical analysis, the sham vs DED groups were compared for each condition using the Kolmogorov–Smirnov test, followed by the parametric *t*-test, using GraphPad Prism version 7.00 (GraphPad Software, La Jolla, CA, USA)

Corneal nerve responsiveness was evaluated at d21 by multi-unit extracellular recording of ciliary nerve fiber activity in ex vivo eye preparations from sham and DED animals. An electrophysiological trace illustrating the extracellular activity of the ciliary nerve fibers for the various heat ramps from 32 to 20°C and 32 to 40°C, with a time scale of 5 s, is shown in Fig. 3a, and histograms representing the number of impulses per second from sham and DED mice are shown in Fig. 3b. The ongoing activity was 54.35% higher in the DED than sham mice at 32°C (59.96 ± 4.77 imp/s vs. 92.55 ± 6.61 imp/s, *P* < 0.001, Fig. 3b). Furthermore, we perfused the cornea with superfusion saline solution at 20°C to activate corneal cold receptors. The frequency of evoked activity was 36.9% higher for the DED than sham mice at 20°C (97.65 ± 5.20 imp/s vs. 133.67 ± 8.09 imp/s, *P* < 0.001, Fig. 3b). The cornea was finally perfused with superfusion saline solution at 40°C to activate polymodal nociceptors. The frequency of firing was 48.4% for the DED than sham mice (64.18 ± 6.80 imp/s vs. 95.24 ± 9.35 imp/s, *P* < 0.01, Fig. 3b). Representative histograms and electrophysiological traces of ciliary nerve-fiber activity in ex vivo eye preparations from sham and DED animals at 20, 32, and 40°C are illustrated in Fig. 3 c–h.

#### Acid stimulation

We next recorded spontaneous activity while perfusing the cornea with a superfusion saline solution at pH 7.4. The spontaneous activity was 73.15% higher for the DED than sham mice (59.96 ± 4.77 imp/s vs. 92.55 ± 6.61 imp/s, *P* < 0.001, Fig. 3i). Then, acid corneal stimulation was induced by lowering the pH of the superfusion saline solution from 7.4 to 6 and then 5. The firing of evoked ciliary nerve activities was 46.64% higher at pH 6 (81.88 ± 4.97 imp/s vs. 120.07 ± 9.86 imp/s, *P* < 0.01, Fig. 3i) and 52.17% higher at pH 5 (78.50 ± 6.11 imp/s vs. 119.45 ± 13.21 imp/s, *P* < 0.01, Fig. 3i) for the DED than sham mice. Representative histograms and electrophysiological traces of ciliary nerve fiber activity in ex vivo eye preparations from sham and DED animals at pH 5, 6, and 7.4 are presented in Fig. 3 j–o.

#### Chemical stimulation: CO_2_

**Fig. 4:**
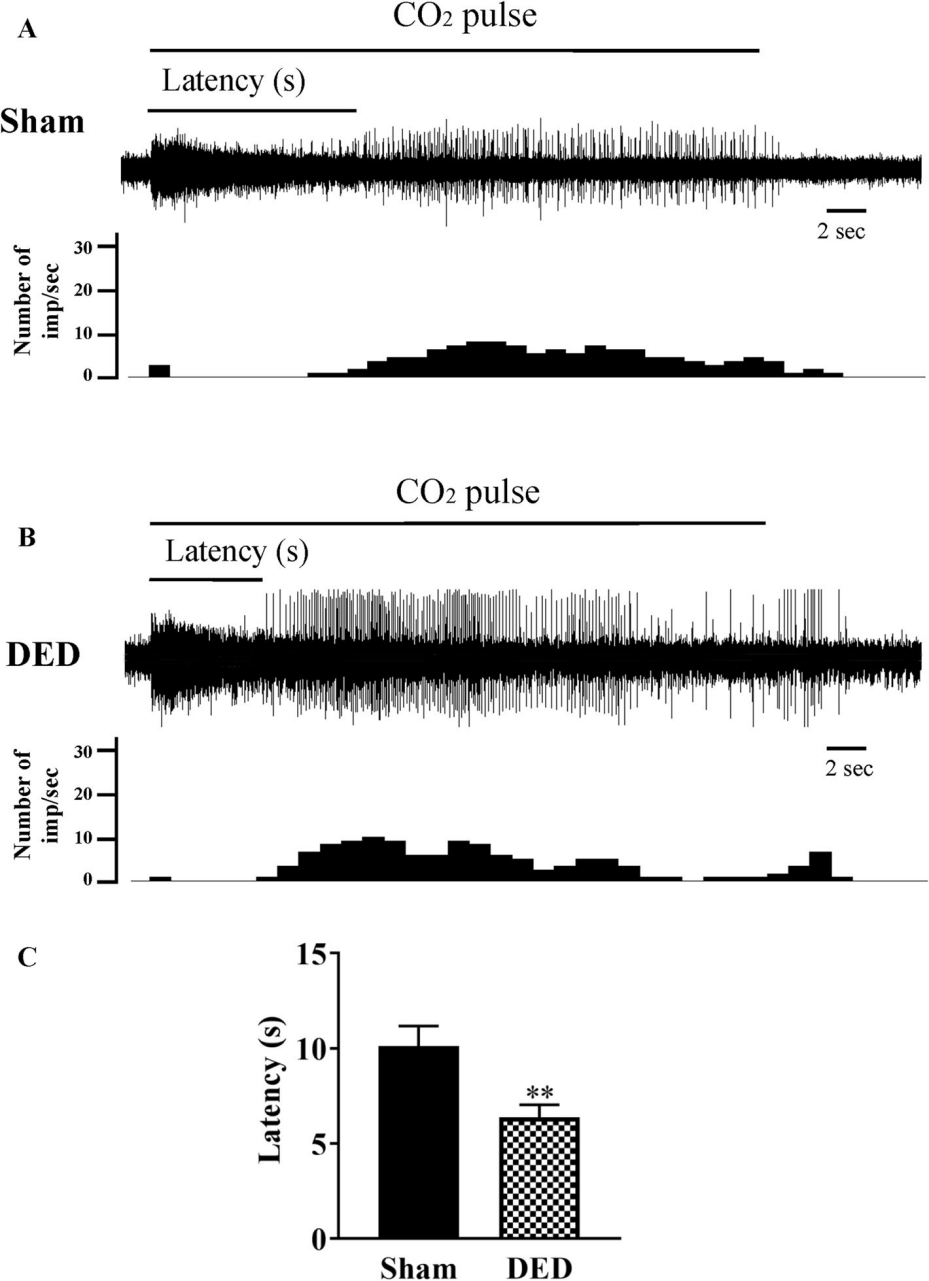
Responsiveness of corneal polymodal nociceptive fibers to CO_2_ stimulation for sham and DED animals. **a**, **b** Electrophysiological traces and histograms illustrating corneal responsiveness to a 30-s CO_2_ pulse. **c** Histogram showing the latency of impulse discharge evoked by CO_2_ for sham and DED animals. Total number of mice = 16. Number of mice included in the analysis =16. Sham and DED animals: *n* = 8 per group. All experiments were conducted on d21. **P < 0.01 relative to sham group. Results are expressed as the mean ± SEM. For statistical analysis, the Kolmogorov–Smirnov test was performed followed by a parametric *t*-test using GraphPad Prism version 7.00 (GraphPad Software, La Jolla, CA, USA)

CO_2_ gas pulses are a commonly used chemical stimulation to activate corneal polymodal nociceptors [27, 31]. Representative electrophysiological traces and histograms illustrating the number of impulses per second from sham and DED mice during a 30s CO_2_ pulse are presented in Fig. 4 a and b. The latency of the impulse discharge evoked by CO_2_ was 36.86% lower for DED than sham mice (10.12 ± 1.03 s vs. 6.39 ± 0.66 s, *P* < 0.01, Fig. 4c).

### Topical treatment with capsazepine decreases the expression of genes related to neuropathic pain and inflammation in the TG of DED animals

We next evaluated the mRNA levels of genes involved in the conduction and modulation of pain in the TG of DED animals relative to that of sham animals and in the TG of DED animals treated with 10 μM capsazepine versus vehicle, taking into consideration that the vehicle hydrates dry corneas and artificial tears are one of the strategies to relieve the symptoms of DED patients.

#### Glial-inflammatory cell communication

**Fig. 5:**
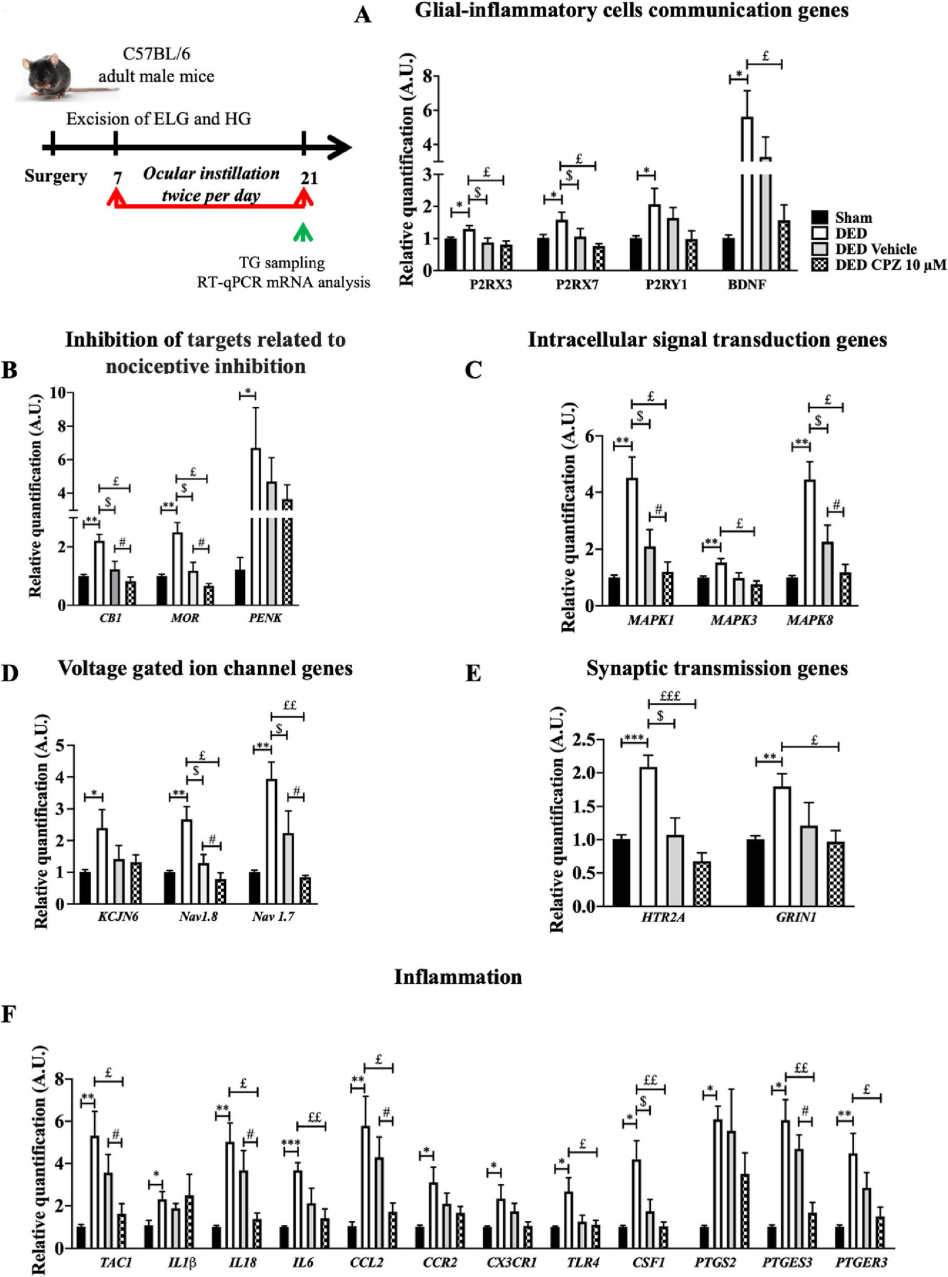
Evaluation of the expression of genes involved in pain conduction and the modulation of pain responses in the TG of untreated sham and DED animals and DED animals treated with 10 μM capsazepine or vehicle on d21. RT-qPCR analysis of ipsilateral TG of sham mice, DED mice, and DED mice treated with 10μM capsazepine or vehicle: glial-inflammatory cell communication genes (**a**), inhibition of targets related to nociceptive inhibition (**b**), intracellular signal transduction genes (**c**), voltage-gated ion channel genes (**d**), synaptic transmission genes (**e**), inflammation genes (**f**). Total number of mice = 19. Number of mice included in the analysis = 19. Sham mice, DED mice, and DED ice-treated with 10μM capsazepine or vehicle: *n* =4–5. *P < 0.05, **P < 0.01, ***P < 0.001, ****P < 0.0001 relative to sham animals. $ < 0.05: DED treated with vehicle relative to DED. £ < 0.05, ££ < 0.01, £££ < 0.001: DED treated with capsazepine relative to DED, # *P*<0.05: DED treated with capsazepine relative to DED treated with vehicle. Results are expressed as the mean ± SEM. For statistical analysis, the Kolmogorov–Smirnov test was performed followed by a nonparametric Mann-Whitney test or a parametric *t*-test using GraphPad Prism version 7.00 (GraphPad Software, La Jolla, CA, USA)

Neuronal-glial communication mediated by purinergic signaling participates in chronic pain [32]. Adenosine triphosphate (ATP) has been proposed to activate P2X and P2Y purinergic receptors, contributing to inflammation [33, 34]. Moreover, activation of purinergic receptors contributes to acute nociception and the maintenance of nociceptive sensitivity [35]. P2RX3, P2RX7, and P2RY1 mRNA levels were 293%, 54.9%, and 103.96% higher in the TG of DED than sham mice, respectively (P2RX3 1.00 ± 0.03 vs. 1.30 ± 0.11, P2RX7 1.02 ± 0.10 vs. 1.58 ± 0.23, P2RY1 1.01 ± 0.07 vs. 2.06 ± 049, *P* < 0.05, Fig. 5a). There was no difference in P2RX3, P2RX7, or P2RY1 mRNA expression between DED animals treated with 10 μM capsazepine and those treated with the vehicle group (P2RX3 0.88 ± 0.14 vs. 0.81 ± 0.11, P2RX7 1.10 ± 0.25 vs. 0.77 ± 0.074, P2RY1 1.71 ± 0.79 vs. 0.92 ± 0.78, *P* > 0.05, Fig. 5a). However, administration of either capsazepine or vehicle resulted in lower P2RX3 and P2RX7 mRNA expression than in untreated DED animals (P2RX3: DED animals 1.30 ± 0.11 vs. vehicle group 0.88 ± 0.14 vs. 10 μM capsazepine 0.81 ± 0.11, P2RX7: DED animals 1.58 ± 0.23 vs. vehicle group 1.10 ± 0.25 vs 10 μM capsazepine group 0.77 ± 0.074, *P* < 0.05, Fig. 5a).

Brain-derived neurotrophic factor (BDNF) plays a functional role in peripheral and central sensitization processes. Its expression is induced by ATP via purinergic receptors [36]. Here, BDNF mRNA levels were 465% higher in DED than sham animals (1.01 ± 0.09 vs. 5.62 ± 1.52, *P* < 0.05, Fig. 5a). However, chronic treatment with capsazepine did not alter BDNF mRNA expression in DED animals relative to that in the vehicle group (3.30 ± 1.21 vs. 1.60 ± 0.47, *P* > 0.05, Fig. 5a). Nevertheless, BDNF mRNA expression was lower in DED animals treated with 10 μM capsazepine than those that were not (5.62 ± 1.52 vs. 1.60 ± 0.47, *P* < 0.05, Fig. 5a).

#### Targets related to nociceptive inhibition

The mu opioid receptor (MOR), cannabinoid receptor 1 (CB1), and proenkephalin (PENK) are known to inhibit nociceptive transmission [37–39]. The mRNA levels of MOR, CB1, and PENK were 149%, 121%, and 149% higher in the TG of DED animals than those of sham animals, respectively (MOR 1.00 ± 0.05 vs. 2.49 ± 0.33, CB1 1.00 ± 0.05 vs. 2.21 ± 0.21, *P* < 0.01, and PENK 1.00 ± 0.05 vs. 2.49 ± 0.33, *P* < 0.05, Fig. 5b).

CB1 and MOR mRNA expression of DED animals treated with 10 μM capsazepine was 55.93% and 44% lower than that of the vehicle group, respectively (CB1 1.21 ± 0.27 vs. 0.82 ± 0.24, *P* < 0.05; MOR 1.21 ± 0.29 vs. 0.66 ± 0.07, *P* < 0.05; Fig. 5b), whereas there was no significant difference in PENK mRNA expression between the two groups (5.46 ± 2.66 vs. 2.80 ± 0.55, *P* > 0.05, Fig. 5b). Moreover, ocular instillation with capsazepine or vehicle resulted in lower CB1 and MOR mRNA levels in treated than untreated DED animals (CB1: DED animals 2.21 ± 0.21 vs. vehicle group 1.21± 0.27 vs. 10 μM capsazepine group 0.82 ± 0.24, *P* < 0.05; MOR: DED animals 2.49 ± 0.33 vs. vehicle group 1.21 ± 0.29 vs. 10 μM capsazepine 0.66 ± 0.07, *P* < 0.05; Fig. 5b).

#### Intracellular signal transduction

Mitogen-activated protein kinases (MAPKs) are essential mediators of signal transduction, and their activation contributes to hypersensitivity to pain [40]. MAPK 1, 3, and 8 mRNA levels were 342%, 49%, and 340% higher in the TG of DED than sham animals, respectively (MAPK1 1.02 ± 0.07 vs. 4.51 ± 0.73, MAPK3 1.02 ± 0.04 vs. 1.52 ± 0.14, *P* < 0.01, and MAPK 8 1.01 ± 0.06 vs. 4.45 ± 0.62, *P* < 0.001; Fig. 5c).

Chronic instillation of DED animals with 10 μM capsazepine resulted in MAPK1 and MAPK8 mRNA levels that were 43% and 47% lower, respectively, than those in mice treated with vehicle (MAPKI 2.09 ± 0.58 vs. 1.20 ± 0.33 and MAPK8 2.26 ± 0.58 vs. 1.18 ± 0.28, *P* < 0.05; Fig. 5c). MAPK3 mRNA expression was not significantly different in DED animals treated with 10 μM capsazepine relative to that of the vehicle group (0.86 ± 0.26 vs. 0.60 ± 0.17, *P* > 0.05, Fig. 5c). In addition, corneal instillation with capsazepine or vehicle resulted in lower MAPK1 and MAPK8 mRNA levels in the treated than untreated DED animals (MAPKI: DED animals 4.51 ± 0.73 vs. vehicle 2.09 ± 0.58 vs. 10 μM capsazepine 1.20 ± 0.33 and MAPK8: DED animals 4.45 ± 0.62 vs. vehicle 2.26 ± 0.58 vs. 10 μM capsazepine 1.18 ± 0.28, *P* < 0.05, Fig. 5c).

#### Voltage-gated ion channels

Electrical excitation of peripheral somatosensory nerves is controlled by potassium and sodium ion channels [41, 42]. We found potassium channel 2 (KCJN6) mRNA levels to be 136.63% higher in DED than sham animals (KCJN6 1.01 ± 0.07 vs. 2.39 ± 0.58, *P* < 0.05, Fig. 5d). In addition, sodium channel 1.8 (Nav1.8) and 1.7 (Nav1.7) mRNA levels were 166% and 293% higher in DED than sham animals, respectively (Nav1.8 1.00 ± 0.05 vs. 2.66 ± 0.40, Nav1.7 1.00 ± 0.06 vs. 3.93 ± 0.53, *P* < 0.01, Fig. 5d).

Capsazepine treatment resulted in Nav1.8 and Nav1.7 mRNA levels that were 40% and 62% lower, respectively, than in animals treated with vehicle (Nav1.8 1.29 ± 0.27 vs. 0.78 ± 0.19, Nav1.7 2.23 ± 0.70 vs. 0.84 ± 0.05, *P* < 0.05, Fig. 5d). We observed no significant difference of KCJN6 mRNA expression in the TG of DED animals treated with 10 μM capsazepine relative to those treated with vehicle (KCJN6 1.13 ± 0.21 vs. 1.34 ± 0.17, *P* > 0.05, Fig. 5d). However, both treatments resulted in lower Nav1.8 and Nav1.7 mRNA levels in both groups than in untreated DED animals (Nav1.8: DED 2.66 ± 0.40 vs. vehicle group 1.29 ± 0.27 vs. 10 μM capsazepine 0.78 ± 0.19, *P* < 0.05; Nav1.7: DED 3.93 ± 0.53 vs. vehicle 2.23 ± 0.70, *P* < 0.05 vs. 10 μM capsazepine 0.84 ± 0.05, *P* < 0.01; Fig. 5d).

#### Synaptic transmission

Increased electrical excitation increases synaptic transmission. We investigated serotonin and glutamate receptor expression in the TGs of DED and sham animals. The levels of 5-hydroxytryptamine receptor 2A (HTR2A) and glutamate (NMDA) receptor subunit zeta-1 (GRIN1) mRNA were 109% and 79% higher in the TG of DED than sham animals, respectively (HTR2A 1.00 ± 0.06 vs. 2.09 ± 0.17, *P* < 0.001; GRIN1 1.00 ± 0.05 vs. 1.79 ± 0.18, *P* < 0.01; Fig. 5e).

Chronic ocular treatment with capsazepine did not alter HTR2A and NMDA mRNA levels in DED animals relative to the vehicle group (HTR2A 1.10 ± 0.25 vs. 0.68 ± 0.13, GRIN1 0.96 ± 0.05 vs. 0.97 ± 0.16, *P* > 0.05; Fig. 5e). HTR2A mRNA levels were, however, lower in capsazepine- and vehicle-treated than untreated DED animals (HTR2A: DED 2.09 ± 0.17 vs. vehicle 1.10 ± 0.25, *P* < 0.05 vs. 10 μM capsazepine 0.68 ± 0.13, *P* < 0.001, Fig. 5e). Moreover, capsazepine treatment resulted in lower NMDA mRNA levels in DED animals treated with 10 μM capsazepine than in untreated DED animals (GRIN1 1.79 ± 0.18 vs. 0.97 ± 0.16, *P* < 0.05, Fig. 5e).

#### Inflammation

The mRNA levels of prostaglandin-endoperoxide synthase 2 (PTGS2), also known as cyclooxygenase-2 (COX-2), prostaglandin E synthase 3 (PTGES3), and prostaglandin E receptor 3 (PTGER3), were 502%, 500%, and 342% higher, respectively, in the TG of DED than sham animals (PTGS2 1.01 ± 0.07 vs. 6.08 ± 0.62, PTGES3 1.01 ± 0.09 vs. 6.05 ± 0.98, *P* < 0.05, and PTGER3 1.01 ± 0.08 vs. 4.47 ± 0.94, *P* < 0.01; Fig. 5f).

Capsazepine treatment resulted in 45% and 57% lower levels of PTGS2 (COX2) and PTGES3 mRNA, respectively, than in the vehicle group (PTGS2 6.39 ± 2.30 vs. 3.51 ± 0.98, *P* > 0.05, and PTGES3 3.98 ± 0.85 vs. 1.68 ± 0.47, *P* < 0.05; Fig. 5f). However, topical capsazepine treatment did not significantly alter IL1β, CCR2, CX3CR1, TLR4, CSF1, or PTGER3 mRNA levels relative to those of the vehicle group (IL1β 1.89 ± 0.07 vs. 1.82 ± 0.06, CCR2 2.13 ± 0.50 vs. 1.62 ± 0.30, CX3CR1 1.67 ± 0.65 vs. 1.01 ± 0.60, TLR4 1.28 ± 0.17 vs. 1.10 ± 0.08, CSF1 1.51 ± 0.61 vs. 0.89 ± 0.30, PTGER3 2.60 ± 1.43 vs. 1.51 ± 0.43, *P* > 0.05; Fig. 5e). Only CSF1 mRNA expression was lower in DED animals treated with the vehicle than in untreated DED animals (CSF1 4.19 ± 0.87 vs. 1.51 ± 0.61 vs. 0.89, *P* < 0.05, Fig. 5e). However, capsazepine treatment of DED mice resulted in lower PTGES3 and PTGER3 mRNA levels than in untreated DED mice (PTGES3 6.05 ± 0.98 vs. 1.68 ± 0.47, *P* < 0.01; PTGER3 4.47 ± 0.94 vs. 1.51 ± 0.43, *P* < 0.05, Fig. 5e).

The levels of tachykinin precursor 1 (TAC1), colony-stimulating factor 1 (CSF1), and toll-like receptor 4 (TLR4) mRNA were also higher in the TG of DED than sham animals, 421%, 315%, and 166%, respectively (TAC1 1.02 ± 0.10 vs. 5.31 ± 1.15, *P* < 0.01; CSF1 1.01 ± 0.06 vs. 4.19 ± 0.87, *P* < 0.05; and TLR4 1.00 ± 0.04 vs. 2.66 ± 0.65, *P* < 0.05; Fig. 5f), as were the levels of interleukin (IL)-1β, IL-18, and IL-6 mRNA, which were 113%, 398%, and 267% higher, respectively, in the DED animals (IL1β 1.08 ± 0.23 vs. 2.30 ± 0.36, *P* < 0.05; IL18 1.01 ± 0.07 vs. 5.03 ± 0.89, *P* < 0.01; and IL6 1.00 ± 0.05 vs. 3.67 ± 0.35, *P* < 0.0001; Fig. 5f). Furthermore, the levels of chemokine (C-C motif) ligand 2 (CCL2), C-C chemokine receptor type 2 (CCR2), and CX3C chemokine receptor 1 (CX3CR1) mRNA were 450%, 207%, and 134% higher, respectively, in the TG of DED animals (CCL2 1.05 ± 0.19 vs. 5.78 ± 1.39, *P* < 0.01; CCR2 1.01 ± 0.07 vs. 3.10 ± 0.71, *P* < 0.05; CX3CR1 1.00 ± 0.05 vs. 2.34 ± 0.64, *P* < 0.05; Fig. 5f).

Moreover, the levels of TAC1 mRNA were 54% lower in DED mice treated with 10 μM capsazepine than those treated with vehicle (3.56 ± 0.85 vs. 1.63 ± 0.46, *P* < 0.05, Fig. 5f), and IL18 and IL6 mRNA levels were also lower by 63% and 74%, respectively (IL18 3.67 ± 0.92 vs. 1.39 ± 0.27, *P* < 0.05 and IL6 5.60 ± 3.20 vs. 1.42 ± 0.43, *P* > 0.05; Fig. 5f). Treatment of DED mice with 10 μM capsazepine also resulted in chemokine CCL2 mRNA levels that were 59% lower than in mice treated with vehicle (4.28 ± 0.96 vs. 1.73 ± 0.39, *P* < 0.05; Fig. 5f). In addition, topical capsazepine administration to DED animals resulted in lower TAC1, IL18, IL6, CCL2, TLR4, and CSF1 mRNA levels than in untreated DED animals (TAC1 5.31 ± 1.15 vs. 1.63 ± 0.46, *P* < 0.05; IL18 5.03 ± 0.89 vs. 1.39 ± 0.27, *P* < 0.05; IL6 3.67 ± 0.35 vs. 1.42 ± 0.43, *P* < 0.01; CCL2 5.78 ± 1.39 vs. 1.73 ± 0.39, *P* < 0.05; TLR4 2.66 ± 0.65 vs. 1.10 ± 0.21, *P* < 0.05; and CFS1 4.19 ± 0.87 vs. 1.00 ± 0.20, *P* < 0.01; Fig. 5f).

### Pharmacological blockage of TRPV1 reduces corneal nociception and anxiety of DED mice

**Fig. 6:**
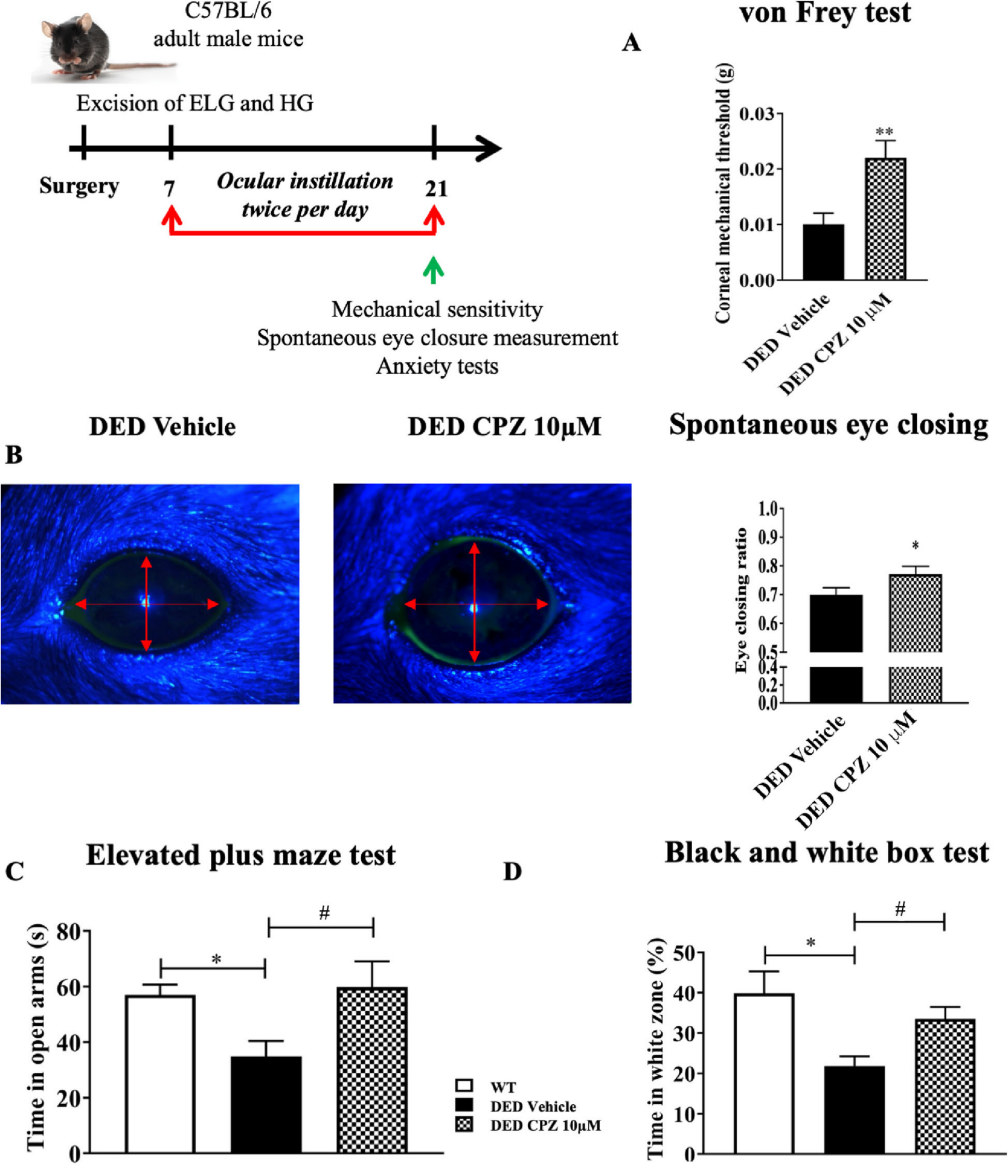
Mechanical allodynia, eye closing ratio, and anxiety of DED animals treated with 10 μM capsazepine or vehicle. **a** Corneal mechanical sensitivity measured with von Frey filaments. **b** Images and quantification of the spontaneous eye closing ratio calculated by measuring the width/height ratio. **c** Time spent in the open arms (s) in the elevated plus maze test. **d** Time spent in the white zone (%) in the black and white box test by DED animals treated with 10 μM capsazepine or vehicle. Total number of mice = 20. Number of mice included in the analysis = 20. DED animals treated with vehicle and 10 μM capsazepine: *n* = 10. All tests were conducted on d21. *P < 0.05, **P < 0.01 relative to DED treated with vehicle. Results are expressed as the mean ± SEM. For statistical analysis, the Kolmogorov–Smirnov test was performed followed by a parametric *t*-test (von Frey and spontaneous eye closure ratio) or the nonparametric Kruskal-Wallis test (elevated plus maze test and black and white test) using GraphPad Prism version 7.00 (GraphPad Software, La Jolla, CA, USA)

We next investigated the impact of topical treatment of capsazepine on corneal mechanical allodynia associated with DED. The mechanical threshold was 120% higher in DED animals treated with 10 μM capsazepine than those receiving vehicle (vehicle 0.010 ± 0.002 vs. 10 μM capsazepine 0.022 ± 0.003, *P* < 0.01; Fig. 6a). The eye-closing ratio was also 9.86% higher for DED animals treated with 10 μM capsazepine than those treated with vehicle (vehicle 0.71 ± 0.02 vs. 10 μM capsazepine 0.78 ± 0.02, *P* < 0.05; Fig. 6b). Next, we investigated the effect of topical capsazepine on anxiety-like behavior associated with DED. Animals treated with capsazepine spent 94% more time in the open arms than those treated with vehicle (control animals 59.01 ± 3.31 s vs vehicle 30.91 ± 3.54 s vs. 10 μM capsazepine 59.83 ± 9.27 s, *P* < 0.05; Fig. 6c). The time spent in the open arms by control animals (WT) was significantly different from that of DED animals treated with vehicle but not from that of the capsazepine group. Moreover, the black and white box test showed capsazepine treatment to increase the time spent in the white zone by DED animals by 54% relative to those receiving vehicle (control animals 39.83 ± 5.47 s vs vehicle 21.80 ± 2.37 s vs. 10 μM capsazepine 33.50 ± 2.97 s, *P* < 0.05; Fig. 6d). The time spent in the white zone by control animals was significantly different from that of DED animals treated with vehicle but not from those receiving topical 10 μM capsazepine.

### Topical treatment with capsazepine reduces corneal nociceptor sensitization in DED animals

**Fig. 7:**
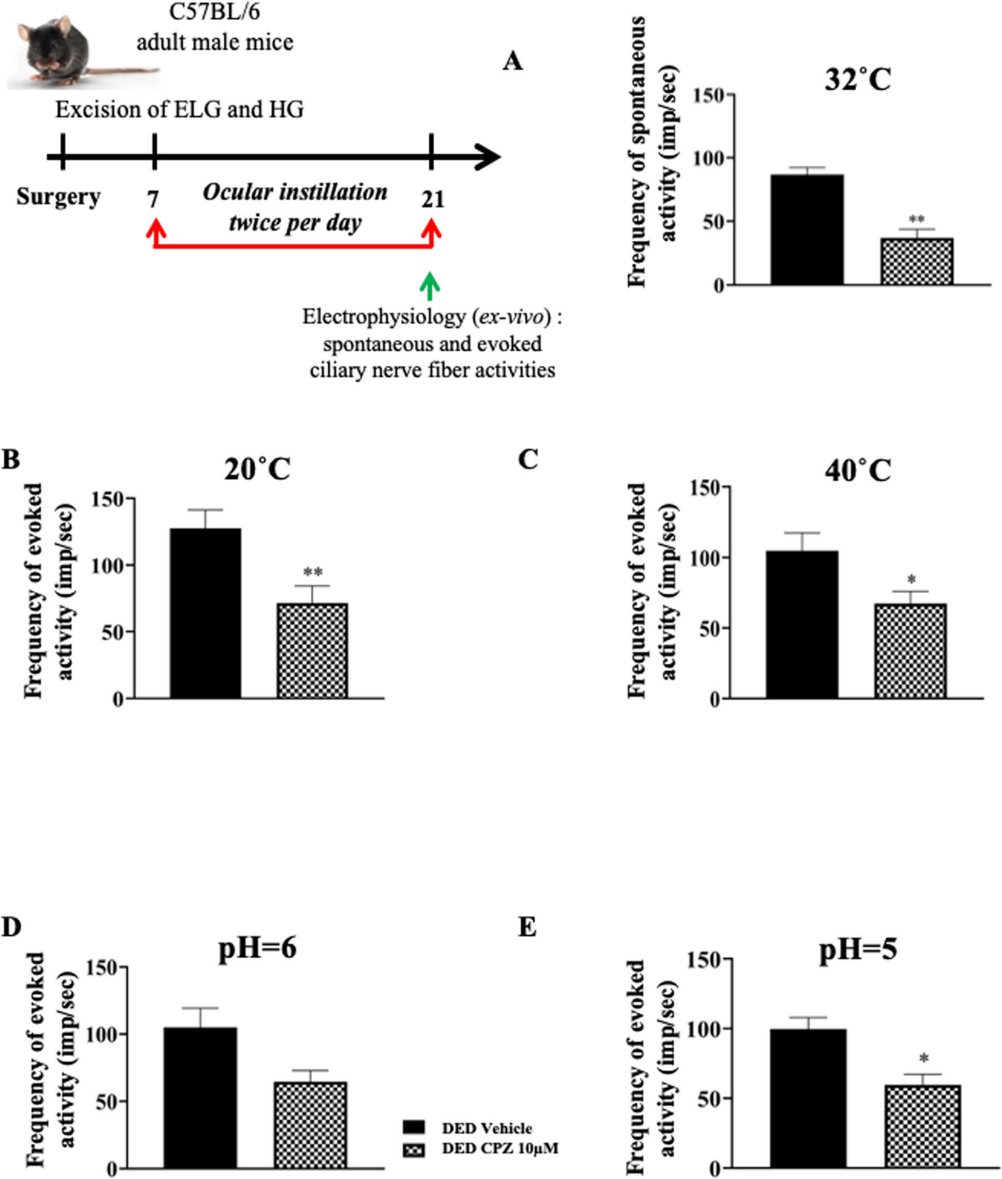
Ex vivo evaluation of spontaneous and evoked activity of the ciliary nerve in DED animals treated with 10μM capsazepine or vehicle. Histograms show the mean value of the frequency activity of the ciliary nerve at (**a**) 32°C and pH 7.4, (**b**) 20°C, (**c**) 40°C, (**d**) pH 6, and (**e**) pH 5 for DED animals treated with 10 μM capsazepine or vehicle. Total number of mice = 20. Number of mice included in the analysis = 20. DED animals treated with vehicle and 10 μM capsazepine: *n* = 10. All experiments were conducted on d21. *P < 0.05, **P < 0.01 relative to DED animals treated with vehicle. Results are expressed as the mean ± SEM. For statistical analysis, the Kolmogorov–Smirnov test was performed followed by a parametric *t*-test using GraphPad Prism version 7.00 (GraphPad Software, La Jolla, CA, USA)

DED animals were treated twice per day from d7 through d21 with 10 μM capsazepine and compared to DED animals treated with vehicle to determine the involvement of the TRPV1 channel in corneal nerve hyperexcitability. Spontaneous activity was 57% lower at 32°C for DED mice treated with 10 μM capsazepine than those treated with vehicle (vehicle 86.78 ± 5.57 imp/s vs. 10 μM capsazepine 37.10 ± 6.83, *P* < 0.01; Fig. 7a). Next, we evaluated the effect of 10 μM capsazepine on corneal nerve activity triggered by heat and cold. Evoked activity was 35% and 44% lower at 20°C and 40°C, respectively for DED mice treated with 10 μM capsazepine than those treated with vehicle (20°C: vehicle 127.71 ± 13.52 vs. 10 μM capsazepine 71.41 ± 12.85, *P* < 0.01, Fig. 7b and 40°C: vehicle 104.57 ± 12.74 vs. 10 μM capsazepine 67.20 ± 8.59, *P* < 0.05, Fig. 7c). We also evaluated the effect of 10 μM capsazepine on corneal nerve activity in response to acid stimulation. Evoked activity was 38% and 40% lower at pH 6 and 5, respectively for DED mice treated with 10 μM capsazepine than those treated with vehicle (pH 6: vehicle 105.01 ± 14.33 vs. 10 μM capsazepine 64.60 ± 8.29, *P* > 0.05, Fig. 7d and pH 5 99.71 ± 8.16 vs. 10 μM capsazepine 59.61 ± 7.56, *P* < 0.05, Fig. 7e).

## Discussion

Despite the high prevalence of DED, the underlying mechanisms of this ocular surface disease are not fully understood. Here, we hypothesized that repeated instillations of a TRPV1 antagonist (capsazepine) could alleviate ocular pain syndrome in severe DED mice. Capsazepine is a synthetic analog of capsaicin that acts as a TRPV1antagonist, leading to suppression of Ca^2+^influx [43]. In addition, it can target other TRP channels, such as TRPV-4 and TRPM-8. Docherty et al. reported that capsazepine non-specifically blocks voltage-activated calcium channels [44]. Kistner et al. found that capsazepine can also exhibit inhibitory effects on colitis via the modulation of TRPA1 [45]. This molecule has been extensively used in spinal and trigeminal pain studies to specifically block the TRPV1 channel [14, 43, 46–48]. A major finding of our work is that repeated instillation of capsazepine has beneficial effects on DED mice: it decreases (1) corneal polymodal responsiveness, (2) the upregulation of genes related to inflammation and pain in the TG, and (3) corneal hypersensitivity and anxiety-like behavior associated with persistent DED.

Here, we used our recently published model of severe DED obtained by excision of the ELG and HG from adult male mice [22]. We first carried out a comparative study of the behavior and electrophysiological responses related to the present study between male and female DED and sham-operated adult mice, which showed no sex-based differences (data not shown).

All animal procedures were performed in strict accordance with institutional guidelines for the care and use of experimental animals, respecting the 3Rs for the use of animals. The removal of two different functional glands (aqueous and lipid) reduces tear production by 97% [22] and closely mimics both aqueous-deficient DED by excision of the ELG and evaporative DED by excision of the HG, which produces an oily, lipid-enriched secretion. The severe DED induced by this model may be considered to be a limitation. However, our DED model, along with being associated with increased ocular nociception, is chronic and does not require repeated injections of chemical solutions with neurotropic activity (such as scopolamine) or the placing of mice in a desiccative environment, which could be stressful for them.

During inflammation, it has been demonstrated that reducing the extracellular pH enhances pain [49]. Indeed, the corneal peripheral free terminals of Aδ and C-fibers are able to detect acidosis through two polymodal channels, TRPV1 and ASICs [50, 51]. After TRPV1 and ASIC depolarization, cationic currents generate action potentials, leading to pain [52, 53]. We demonstrate that chronic DED induces sensitization of corneal polymodal nerves in response to a CO_2_ gas jet and acid stimulation (pH 5 and 6) using an electrophysiological ex vivo recording of ciliary nerve fiber activity. Such corneal nerve sensitization can be explained by higher TRPVI and ASIC mRNA expression in the ophthalmic branch of the TG of DED animals. It has been established that only 50% of all CO_2_-activated fibers respond to stimulation by capsaicin (a TRPV1 agonist) in the cat cornea [54, 55]. Thus, we suggest that both TRPV1 and ASIC channels are involved in the response to acid stimulation in DED.

Thermal hypersensitivity is common in inflammation and nervous system damage [56]. Both TRPV1 and TRPA1 polymodal channels are known to be activated by acute heat [57]. Interestingly, the firing of ciliary nerves in DED mice increased with heat stimulation, demonstrating an intensification of polymodal responsiveness to heat. This finding is in accordance with higher mRNA levels of both TRPA1 and TRPV1 channels in the TGs of DED animals. Thus, we provide further evidence of polymodal nociceptor sensitization at the level of the cornea and peripheral nervous system in DED animals. Moreover, it is known that activation of polymodal nociceptors evokes a burning pain [10]. This is in accordance with the increased nocifensive behaviors observed after topical application of capsaicin in DED animals. We suggest that DED-induced polymodal sensitization may be a mechanism responsible for the enhanced pain and burning sensations classically observed in DED patients [58].

On the other hand, our data show that a TRPV1 antagonist (capsazepine) decreases polymodal responsiveness to acid and heat stimulation, in accordance with capsazepine-induced reduction of polymodal nociceptor responses to acid stimulation in an allergic eye model [19]. TRPV1 has high Ca^2+^ permeability, and it has been reported that heat stimulation increases calcium concentrations via TRPV1; such an increase was blocked with capsazepine [59]. This finding explains the effectiveness of specific TRPV1 antagonism to heat and acid stimulation in DED animals.

Belmonte et al. showed that ongoing ciliary nerve fiber activity results mostly from cold nerve fiber activation, which represents half of corneal sensory neurons [11, 31]. Thus, the increase in spontaneous ciliary nerve fiber activity observed in DED animals may result from the triggering of corneal cold nociceptors, as already reported for DED guinea pigs [13]. Interestingly, despite the insensitivity of TRPV1 to cold stimulation, capsazepine blunted the DED-induced increase of ciliary nerve firing to cold stimulation. It was recently demonstrated that overexpression of TRPV1 in TRPM-8^+^ cold-sensing fibers causes cold allodynia in DED mice obtained by extra orbital gland excision [20]. Thus, we suggest that capsazepine decreases ongoing and stimulated ciliary activity by acting on cold nociceptor responsiveness.

Mice in our DED model, characterized by a decrease in corneal intraepithelial nerve endings, develop increased mechanical sensitivity, as previously reported [22]. Such mechanical hypersensitivity has also been found in an aqueous tear deficiency model of DED in the rat [60] and in mouse models of corneal injury [27].

A reduction in the density of corneal intraepithelial nerves has been reported in several DED models [61–64] and is associated with decreased mechanical sensitivity. In the clinic, studies on patients with DED or neuropathic pain symptoms have shown reduced corneal nerve density associated with decreased sensitivity to mechanical, thermal, and chemical stimuli relative to controls [65], whereas others have shown increased mechanical sensitivity [66]. These contrasting observations from preclinical and clinical studies may be related to varying severity and differing etiology (inflammatory vs neuropathic) of patients, as well as to different models and methods used to measure corneal mechanical sensitivity in preclinical and clinical studies.

Mechano- and polymodal nociceptors respond to mechanical stimulation [8]. Moreover, capsaicin-induced mechanical allodynia in skin is mediated via Piezo-2 [67]. We found that Piezo-2 mRNA levels were not upregulated, unlike those of TRPV1, in the TG of DED mice relative to sham animals. In addition, topical capsazepine administration decreased corneal mechanical allodynia in DED animals, suggesting key involvement of polymodal nociceptors in the mechanical allodynia associated with DED. In a recent study, Fernández-Trillo et al. demonstrated that Piezo2 channels are present in the cell body and nerve endings of corneal neurons [68]. They used several modalities and multimodal approaches to show that Piezo2 channels contribute to mechanical sensitivity in sensory nerve endings of pure corneal mechanoreceptors and polymodal nociceptors. Of note, this study did not evaluate the expression of Piezo2 in the cornea or TG of DED mice. Here, we quantified the expression of Piezo2 mRNA by the RNA scope in situ hybridization technique throughout the ophthalmic branch of the TG and found Piezo2 expression to remain unchanged in the state of DED. However, polymodal nociceptors (known to express TRPV1 and to a lesser extent Piezo 2) are also activated by mechanical stimuli. Based on the results of Fernández-Trillo et al., demonstrating that Piezo2 contributes to the mechanical sensitivity in sensory nerve endings of polymodal nociceptors, and the higher expression of TRPV1 in TG in our preclinical DED model, our data suggest that topical treatment with capsazepine reduces (without completely blunting) the mechanical hypersensitivity associated with chronic DED. Such an effect may be mediated primarily by TRPV1 expressed by corneal nociceptors, but the participation of Piezo2 in such mechanical hypersensitivity cannot be excluded.

Numerous studies have demonstrated that pain is modulated by endogenous (enkephalins, endorphins, and dynorphins) opioids and the cannabinoid system [37–39]. Here, we observed that DED induced a significant increase in CB1, MOR, and Penk expression in ipsilateral TG (relative to the sham group); such an increase may be related to sensitization of the corneal nociceptors in DED animals. We previously found higher expression of MOR in corneal nerve fibers and trigeminal sensory neurons in a mouse model of inflammatory corneal pain [69]. In addition, repeated topical ocular administration of DAMGO (a MOR-selective ligand) strongly reduced both mechanical (von Frey) and chemical (capsaicin challenge) corneal hypersensitivity. Topical DAMGO administration also reversed the elevated spontaneous activity of the ciliary nerve and responsiveness of corneal polymodal nociceptors associated with inflammatory ocular pain [69].

Nociceptor sensitization to inflammatory stimuli is a signal to protect the tissue from their harmful consequences. Much data demonstrate that nociceptive neurons express receptors for immune cell-derived inflammatory mediators [70, 71]. Thus, during inflammation, these mediators modify nociceptor responsiveness, notably by inducing phosphorylation of ligand-gated channels, i.e., TRPV1 and TRPA1, leading to increased neuronal firing [72, 73]. Inflammation is the core mechanism of DED, and we recently reported inflammatory responses in the TG from DED mice [22]. Here, we extend our knowledge by providing a molecular signature of TG during persistent DED. Our data show significant upregulation of the mRNA levels of the immune receptors and inflammatory mediators IL1β, IL18, IL6, CCL2, cyclo-oxygenase enzymes, prostaglandins, CX3CR1, CCR2, and TLR4 in the TG of DED animals. These receptors and inflammatory mediators are known to be linked to the pathophysiology of pain [74–76]. Thus, we suggest that these upregulated inflammatory markers may activate peptidergic polymodal nociceptor terminals and stimulate their intracellular signaling pathways. This hypothesis is corroborated by the upregulation of MAPK mRNA levels in the TG of DED animals. Overall, this cascade of events may lead to the excitation of peripheral terminals and the spread of nociceptive messages, which are controlled by potassium and sodium ion channels [41, 42]. The overexpression of mRNA for these channels in the TG could also explain the increase of ciliary nerve firing observed in DED animals.

Additionally, glutamatergic synapses modulate excitatory neurotransmission in nociceptive pathways [77]. The upregulation of glutamate receptor mRNA expression suggests higher nociceptive transduction in the TG of DED animals. Moreover, it has been demonstrated that neuronal–glial communication through purinergic signaling is involved in chronic pain [32]. P2RX3 and P2RX7 also increase pain, whereas P2RY1 decreases its intensity [32, 78]. The upregulation of P2RX3, P2RX7, and P2RY1 mRNA levels observed in the TG of DED animals highlights enhanced neuronal–glial communication in chronic DED, as we previously reported [22].

Capsazepine has been reported to reduce macrophage, eosinophil, and proinflammatory cytokine levels in a chronic asthma model [79]. These anti-inflammatory properties can explain the decrease of inflammatory markers observed in the TG of DED animals chronically treated with topical capsazepine. Moreover, substance P (SP) is encoded by the Tac1 gene and synthesized by C and Aδ nociceptive primary sensory neurons. Once depolarized, corneal polymodal nociceptor neurons release pro-inflammatory neuropeptides, leading to neurogenic inflammation [31, 80]. Ang et al. demonstrated the anti-inflammatory effects of capsazepine, showing it to reduce SP production and proinflammatory molecule levels in a model of polymicrobial sepsis [81]. Thus, the decrease in DED-induced TAC1 mRNA levels in the TG of DED animals by capsazepine highlights a reduction in neurogenic inflammation and the excitation of peripheral free terminals in DED animals. This hypothesis is supported by the decrease of sodium ion channel and MAPK mRNA levels in the TG of DED animals treated with capsazepine.

Preclinical and clinical studies have reported comorbidity between DED and anxiety [82–84]. Thus, we used two behavioral tests (elevated plus maze and black and white tests) and show that DED mice develop anxiety-like behaviors that correlate with the neuronal activation observed in the amygdala, thus linking nociceptive responses with the emotional aspects of pain [85]. These findings highlight the neuropsychiatric impact of chronic DED on these animals, which caused pain and anxiety. Moreover, through this original approach, we showed that DED animals treated with capsazepine spent more time in the anxious zones of both behavioral tests than untreated DED mice, correlating with the lower level of anxiety-related behavior observed in TRPV1 knockout mice [86] and highlighting an anxiolytic effect of capsazepine in DED animals.

## Conclusion

In conclusion, our results provide new arguments on the pharmacological effectiveness of TRPV1 antagonist instillation against DED-induced sensory abnormalities and anxiety, opening a new avenue for the repositioning of this class of molecule as a potential analgesic treatment of patients suffering from chronic DED.

## Data Availability

The datasets analyzed during this study are available from the corresponding author upon reasonable request.

## Abbreviations

AP: Action potential
ASICs: Acid-sensing ion channels
ATP: Adenosine triphosphate
BDNF: Brain-derived neurotrophic factor
CPZ: Capsazepine
CB1: Cannabinoid receptor 1
CCL2: Chemokine (C-C motif) ligand 2
CCR2: C-C chemokine receptor type 2
COX-2: Cyclooxygenase-2
CSF1: Colony-stimulating factor 1
CX3CR1: CX3C chemokine receptor 1
DED: Dry eye disease
ELG: Extraorbital lacrimal gland
GAPDH: Glyceraldehyde 3-phosphate dehydrogenase
GRIN1: Glutamate [NMDA] receptor subunit zeta-1
HG: Harderian gland
HTR2A: 5-Hydroxytryptamine receptor 2A
imp: Impulse
i.p.: Intraperitoneal
KCJN6: Potassium channel 2
IL1β: Interleukin-1β
IL6: Interleukin-6
MAPK: Mitogen-activated protein kinase
MOR: Mu opioid receptor
mRNA: Messenger RNA
Nav1.7: Sodium channel 1.7
Nav1.8: Sodium channel 1.8
PENK: Proenkephalin
Piezo-2: Piezo-type mechanosensitive ion channel component 2
P2RX: Purinergic receptor X
P2RY: Purinergic receptor Y
PTGS2: Prostaglandin-endoperoxide synthase 2
PTGES3: Prostaglandin E synthase 3
PTGER3: Prostaglandin E receptor 3
RT: Room temperature
RT-PCR: Real-time polymerase chain reaction
SEM: Standard error of the mean
TAC1: Tachykinin precursor 1
TBSC: Trigeminal brainstem sensory complex
TBP: TATA-binding protein
TG: Trigeminal ganglion
TLR-4: Toll-like receptor 4
TRPV1: Transient receptor potential vanilloid-1
TRPA1: Transient receptor potential ankyrin 1
TRPM-8: Transient receptor potential melastatin 8
